# Effect of an infection control programme in enteral feeding bacterial contamination in nursing homes

**DOI:** 10.1186/1753-6561-5-S6-P165

**Published:** 2011-06-29

**Authors:** SSK Ho, MMY Tse, MV Boost

**Affiliations:** 1 School of Nursing, Hong Kong, Hong Kong, China; 2 The Hong Kong Polytechnic University, Hong Kong, Hong Kong, China

## Introduction / objectives

This study was to investigate the effectiveness of an Infection Control Programme (ICP) in knowledge and practice of enteral feeding of nursing home staff, and to explore any enteral feed contamination and correlation among nursing home staff, residents and feeding equipment devices.

## Methods

It was a quasi- experimental pretest posttest control study. There were 15 residents and 10 nursing home staff in experimental and control groups respectively. An ICP related to enteral feeding was given to the experimental group. The knowledge and practice in enteral feeding among nursing home staff was measured by questionnaires; and 60 pairs of specimens were taken for bacterial counts and MRSA culture from hands of nursing home staff, enteral feed, flow regulators, feeding tube hubs, residents’ nasopharyngeal swabs and gastric fluid before and after ICP.

## Results

After ICP, only experimental group had significant improvement in knowledge and practice at p<0.05. Also, hands of nursing home staff, tube hubs, residents’ nasal swabs and gastric content were contaminated with >10^4^ CFU/ml in pretest. This contamination was reduced significantly in the experimental posttest at p<0.05 with no change in the control group. Also, there was a significant decrease in MRSA positive cases in the experimental group from 2.1± 1.6 to 0.4± 0.7 with p<0.05. Moreover, highly correlation between contamination sites of positive MRSA in hands, regulators, gastric content, tube hubs and enteral feed was demonstrated at p<0.05. It showed the closely relationship between the contaminated feed and poor hand hygiene.

## Conclusion

The effectiveness of ICP demonstrated by the improvement in experimental enteral feeding contamination conditions. It is strongly to recommend the continuous ICP education.

## Disclosure of interest

None declared.

